# Prevalence of breast cancer in the city of Goiânia, Goiás, Brazil, between 1988 and 2002

**DOI:** 10.1590/S1516-31802011000500005

**Published:** 2011-09-01

**Authors:** Edesio Martins, Ruffo Freitas, Maria Paula Curado, Nilceana Maya Aires Freitas, Carleane Maciel Bandeira Silva, José Carlos Oliveira

**Affiliations:** I MHSc. Epidemiologist, Postgraduate Program on Health Sciences, School of Medicine, Universidade Federal de Goiás (UFG) and Population-Based Cancer Registry of Goiânia, Associação de Combate ao Câncer de Goiás (ACCG), Goiânia, Goiás, Brazil.; II MD, PhD. Gynecologist and Mastologist, Gynecology and Breast Service of Hospital Araújo Jorge, Associação de Combate ao Câncer de Goiás (ACCG), Goiânia, Goiás, Brazil.; III MD, PhD. Senior researcher at the International Prevention Research Institute, Lyon, France, and Population-Based Cancer Registry of Goiânia, Associação de Combate ao Câncer de Goiás (ACCG), Goiânia, Goiás, Brazil.; IV MD, PhD. Radiotherapist, Radiotherapy Service of Hospital Araújo Jorge, Associação de Combate ao Câncer de Goiás (ACCG), Goiânia, Goiás, Brazil.; V Technician at the Population-Based Cancer Registry of Goiânia, Associação de Combate ao Câncer de Goiás (ACCG), Goiânia, Goiás, Brazil.; VI MD, PhD. Head of Head and Neck Surgery Service of Hospital Araújo Jorge and Population-Based Cancer Registry of Goiânia, Associação de Combate ao Câncer de Goiás (ACCG), Goiânia, Goiás, Brazil.

**Keywords:** Breast neoplasms, Epidemiology, Incidence, Prevalence, Brazil, Neoplasias da mama, Epidemiologia, Incidência, Prevalência, Brasil

## Abstract

**CONTEXT AND OBJECTIVE:**

Studies have shown increased prevalence rates for breast cancer, relating to higher incidence, longer survival and breast cancer prevention programs among populations. The aim here was to analyze the annual prevalence of breast cancer in Goiânia over a 15-year period.

**DESIGN AND SETTING:**

This was a cross-sectional study on women with breast cancer diagnosed in Goiânia, Goiás, Brazil, from 1988 to 2002.

**METHODS:**

The breast cancer cases were identified in the database of the Population-Based Cancer Registry of Goiânia. The 15-year period was stratified into three five-year periods. The cases were followed up for five years, and the mortality database was used to exclude deaths. The population of the official census was used as the denominator for rate calculations.

**RESULTS:**

The coefficient of breast cancer prevalence in Goiania was 22.87/100,000 in 1988 and 220.22/100,000 women in 2002. The analyses for periods showed that in the first period, the rate was 19.39/100,000 and that it was 44.79/100,000 in the last period. For the fifteen years analyzed, the prevalence rate for breast cancer was 127.24/100,000 women. The annual percentage change was 27.07 (P < 0.001; 95% confidence interval, CI: 20.79-33.67) from 1988 to 1992 and 9.39 (P < 0.001; 95% CI: 8.52-10.25) from 1992 to 2002.

**CONCLUSION:**

There was an increase in the breast cancer prevalence rate in the city of Goiânia between 1988 and 2002, possibly relating to the improvement in the screening and treatment of breast cancer.

## RESUMO

**CONTEXO E OBJETIVO:**

Estudos têm demonstrado um aumento da taxa de prevalência para o câncer de mama relacionado à maior incidência, maior sobrevida e programas de prevenção do câncer de mama na população. O objetivo foi analisar a prevalência anual de câncer de mama em Goiânia, num período de 15 anos.

**TIPO DE ESTUDO E LOCAL:**

Estudo de corte transversal de mulheres com câncer de mama em Goiânia, Goiás, Brasil, no período de 1988 a 2002.

**METODOS:**

Os casos de câncer de mama foram identificados na base de dados do Registro de Câncer de Base Populacional de Goiânia. Os 15 anos foram estratificados em quinquênios. As pacientes foram seguidas por cinco anos e utilizaram-se os dados da mortalidade para exclusão dos óbitos. Utilizou-se a população do censo oficial como denominador para o cálculo das taxas.

**RESULTADOS:**

O coeficiente de prevalência de câncer de mama em Goiânia foi de 22,87/100000 em 1988 e 220,22/100000 mulheres em 2002. As análises para os períodos evidenciaram que, no primeiro período, a taxa foi de 19,39/100000, e de 44,79/100000 no último período. Nos 15 anos de análise, a taxa de prevalência foi de 127,24/100000 mulheres. A mudança percentual anual foi de 27,07 (P < 0,001; intervalo de confiança, IC 95% 20,79-33,67) de 1988 a 1992 e 9,39 (P < 0,001; IC 95% 8,52-10,25) de 1992 a 2002.

**CONCLUSÃO:**

Houve aumento da taxa de prevalência do câncer de mama na cidade de Goiânia no período de 1988 a 2002, possivelmente relacionado à melhora do rastreamento e do tratamento do câncer de mama.

## INTRODUCTION

Prevalence studies are basic indicators for determining the quantity and impact of diseases among populations over previously established periods.^1,2^ They describe the number of people living with the disease of interest in a particular location or over a certain period. However, with regard to cancer, the definition of prevalence is unclear, since it is difficult to determine what is meant when it is said that an individual has been “cured”.^3^

Determination of the prevalence of a type of neoplasia is a useful tool for planning and administrating health services and programs. For example, the prevalence of the disease needs to be known when aiming to make a certain health service or product available to the population.^1,3^

Among the cancer prevalence rates relating to 25 different topographic sites, increasing breast cancer prevalence rates are being seen in various parts of the world. North America and Europe contribute the highest rates (125.9/100,000 and 106.2/100,000 respectively).^3^

Other studies^2,4-8^ have also shown increased prevalence rates. These increases have been correlated with higher incidence and longer survival^2,7-9^ and with population screening programs, thereby giving rise to higher case prevalence, especially of cases detected in the initial stages.^10^ Such increases made breast cancer the malignant neoplasia of highest prevalence in the year 2002, with 17.9% of the cancer cases worldwide (4.4 million).^11^

Although it is well established that early diagnosis and adequate treatment influence the mortality rates and prevalence of breast cancer, the amount of data available regarding such healthcare tools is small, both for Goiânia and for the whole of Brazil. This makes it difficult to evaluate programs aimed at dealing with malignant breast neoplasms and, especially, makes it impossible to allocate financial and human resources according to the needs of each region.

## OBJECTIVE

This study had the aim of analyzing the prevalence of breast cancer in Goiânia over a 15-year period.

## METHODS

This was a retrospective population-based cross-sectional study on women with malignant breast neoplasms diagnosed in the city of Goiânia, state of Goiás, Brazil.

The cases were identified in the Goiânia Population-Based Cancer Registry database (Registro de Câncer de Base Populacional de Goiânia, RCBP, Goiânia) and the data were gathered between 1988 and 2002.

The patients were followed up for five years from the date of diagnosis. In other words, patients diagnosed in 1988 were followed up until the end of 1992; patients diagnosed in 1989 were followed up until the end of 1993, and so on, until the year 2002. The five-year prevalence was determined for each year of incidence, culminating in determining the total prevalence of breast cancer observed over the 15-year period. These fifteen years were stratified into three five-year periods for comparison purposes, as follows: 1988 to 1992, 1993 to 1997 and 1998 to 2002.

The follow-up was carried out using the mortality information system database supplied by the Municipal Health Department of Goiânia. This base was cross-referenced with the RCBP data in order to correctly exclude patients who died during the observation periods. The population of Goiânia for each period was extracted from the public-domain DATASUS^12^ electronic page of the Brazilian Ministry of Health. The official census was used as the denominator for the rate calculations.

Incidence is the number of new cases arising in a given period in a specified population. This information is collected routinely by cancer registries. It can be expressed as an absolute number of cases per year or as a rate per 100,000 persons per year.

The prevalence rate was defined as the number of existing cases plus the number of new cases over the same period of time, divided by the population for the same period and multiplied by the constant (100,000). To calculate the prevalence rate, according to the period, the denominator used was the number of people per year, calculated from the population at the midpoint of each five-year period, multiplied by the duration of observation (five years). The prevalence is, therefore, the number of people living with the disease in a certain period.

The Poisson regression model was used to determine the annual growth rates of prevalence and incidence over the period from 1988 to 2002. Models that had P-values less than 0.05 with a 95% confidence interval were considered statistically significant.

The eligibility criteria for case inclusion followed the methodology used by RCBP in Goiânia, as previously described.^13-15^ All the breast cancer cases diagnosed each year among women living in the municipality of Goiânia were eligible for inclusion. To avoid bias caused by women from other localities who went to Goiânia seeking treatment, the subjects included in this study needed to have been living in Goiânia for at least six months before they were diagnosed with cancer. The Excel 2003 spreadsheet editor was used to construct the tables and graphs, and the Joinpoint software, version 3.2.0, was used for the regression analysis.^16,17^

## RESULTS

Between 1988 and 2002, 2906 breast cancer cases in Goiânia were identified and 468 deaths due to breast cancer were registered. The incidence of breast cancer changed from 103 cases (3.54%) in 1988 to 362 (12.46%) in 2002, and the prevalence changed from 88 (0.89%) in 1988 to 1281 (12.9%) in 2002.

**Figure 1.**
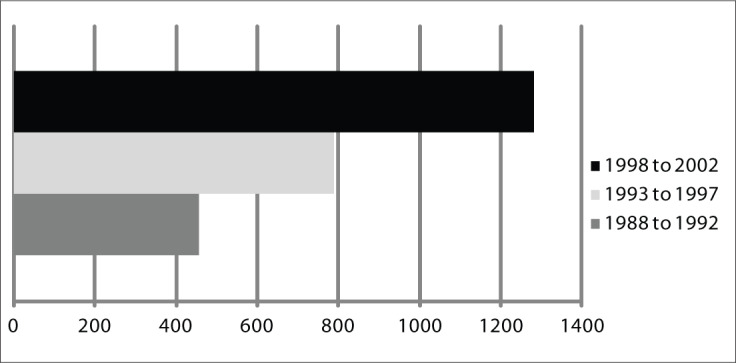
Number of current cases per five-year period in Goiânia, Goiás, Brazil.

The analysis of the five-year periods revealed that, at the end of the first period (1992), there were 456 current cases; at the end of the second period (1997), there were 788 current cases; and at the end of the final five-year period (2002), there were 1281 current cases (Figure 1).

The coefficient of breast cancer prevalence in Goiânia was 22.87/100,000 women in 1988 and 220.22/100,000 women in 2002, with an increase of approximately 197.35/100,000.

**Figure 2.**
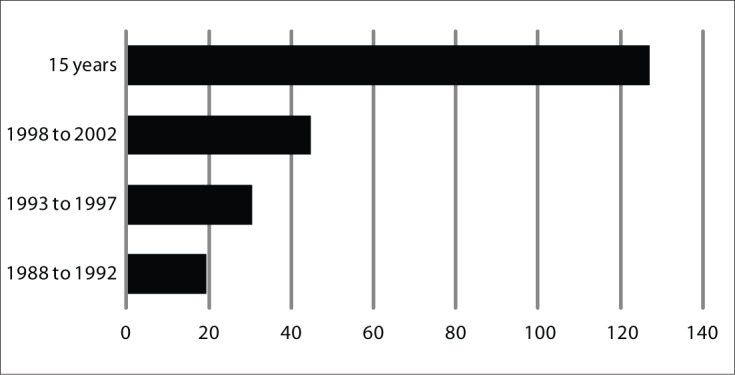
Comparison of breast cancer prevalence between five-year periods and covering 15 years, in Goiânia, Goiás, Brazil.

From the analysis according to five-year periods, it was seen that in the first five-year period, the rate was 19.39/100,000, and it was 44.79/100,000 in the last period. Over the fifteen years analyzed, the prevalence rate for breast cancer was 127.24/100,000 women (Figure 2).

**Figure 3.**
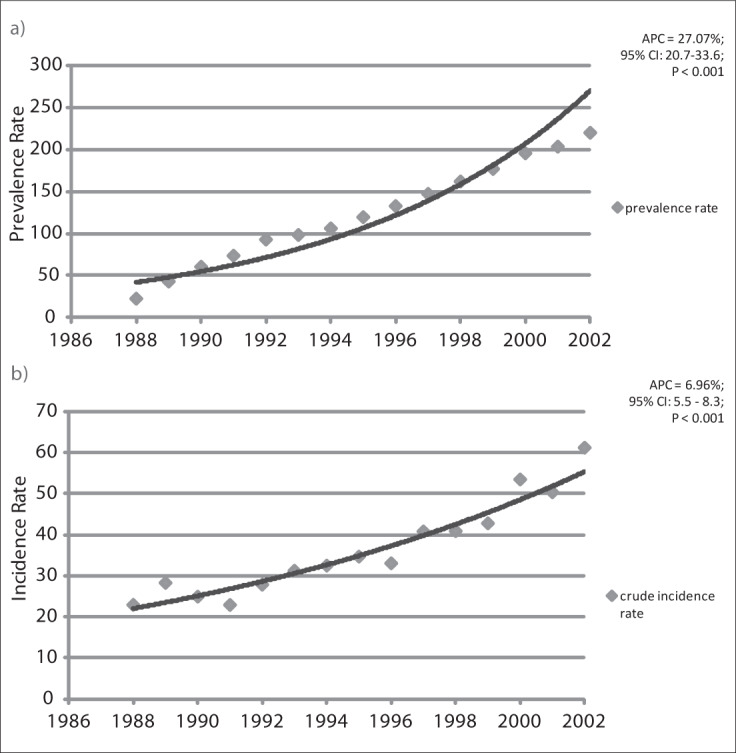
Annual growth trend curves for prevalence (a) and incidence (b) of breast cancer according to the Poisson regression model, between 1988 and 2002 in Goiânia, Goiás, Brazil. APC = annual percentage change; CI = confidence interval.

It was observed that with regard to the prevalence/incidence relationship, there was a significant increase in the prevalence in relation to incidence during the first five-year period, of 27.07% per year (95% confidence interval, CI: 20.79-33.67; P < 0.001). Subsequently, this growth fell to 9.39% (95% CI: 8.52-10.25; P < 0.001) per year during the second and third periods (1992-2002). Regarding incidence, the annual increase was 6.93% (95% CI: 5.5-8.3; P < 0.001) (Figure 3).

**Figure 4.**
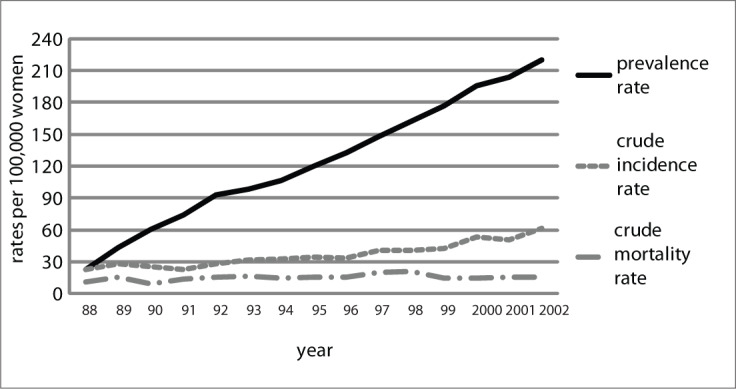
Comparison between the prevalence and incidence rates for breast cancer between 1988 and 2002, in Goiânia, Goiás, Brazil.

Comparison between prevalence, incidence and mortality showed that the increase in prevalence was approximately three times greater than the increase in incidence, while the mortality remained stable over the course of these fifteen years, with a ratio of approximately 3.60 current cases for each incident case in each five-year period (Figure 4).

## DISCUSSION

Models for prevalence estimates are important for planning health programs and resource allocation according to each region's needs. This information is not always available to the bodies responsible for distributing the resources meant for health programs.

Up to a certain point, the breast cancer incidence rate reflects the social pattern, lifestyle, degree of information among the population, existence of screening programs and accessibility in each region.^8,13,14^

Between 1988 and 2002, 2,906 new cases of this malignant type of neoplasia were gathered by RCBP in Goiânia. There was a greater increase from 1996 onwards, which coincided with the implementation of the “Viva Mulher” (“Long Live Women”) breast and cervical cancer prevention programs^13,14,18^ that were implemented by the Ministry of Health. Over the same period, 9,907 women were alive after the diagnosis, with a prevalence of 127/100,000.

The change from 27.07% to 9.39% annual growth in the prevalence observed over the first five years can be explained by the fact that we did not possess information on the preceding years. Thus, when the evaluation was started in 1988, the prevalence was generated considering the incident cases of that year. For the subsequent years, we added together the new and old cases (excluding deaths), until the calculation was stabilized in 1992, at which point there were five complete years of prevalence. Thus, after 1992, it was possible to accurately calculate the AAPC (annual average percentage change).

Despite the Brazilian government's recommendation that mammography should be provided for women aged 40 years and over, the mammography coverage in the state of Goiás is approximately 46% for women in this age group.^19^ Hence, it is far from the recommendation of 70%, which would be necessary for reducing the breast cancer mortality rate.^20^ Therefore, one of the hypotheses raised to explain the increase in breast cancer incidence is that early diagnosis is now occurring, as has been observed in epidemiological studies on this type of neoplasia.^13,14,21^ It can be asked whether this same hypothesis would explain the increase in prevalence observed in the present study, which grew from 22.87/100,000 women in 1988 to 220.22/100,000 women in 2002, with an approximate increase of 197.35/100,000.

In Brazil, the lack of a national breast cancer screening program has led every city to develop its own separate strategies, with the intention of targeting specific populations (opportunistic screening). The main goal of breast cancer screening programs is to promote decreases in the mortality due to this type of neoplasia.^22^ This has not been occurring, since the mortality due to breast cancer in Goiânia remained stable over the 15 years analyzed.^13^ This is different from other countries such as the United States, where the mortality rate has declined by 2.4% among white women and by 1.1% among Afro-American women.^23^

Even so, we believe that mammographic screening has contributed towards early diagnosis, thus leading to an increased incidence rate^11,24^ and, consequently, detection of initial tumors. This has increased survival and stimulated increased prevalence of breast cancer in Goiânia.

Another hypothesis that has been put forward to explain the increased prevalence observed in this study relates to the improvements in treatments. With the advent of new systemic therapies (multidrug therapy) and the development of hormone therapy for adjuvant treatment of breast cancer, changes to the prognostic profile for mortality and recurrence among patients have been obtained, which were only observed after a mean follow-up of 15 years.^25,26^

The neo and adjuvant treatments recommended by the guidelines^27,28^ for breast cancer have contributed towards increases in prevalence due to the increase in life expectancy among younger women and among women between 50 and 69 years of age. However, the same has not occurred for women over 70 years old.^26,29-32^

The reasons for the increase in breast cancer prevalence in Goiânia are not well defined, because of difficulties in establishing the proportions of the contributions between mammographic screening and treatment. We believe that both favor increased survival and reduced mortality among women who have been diagnosed with breast cancer, which would lead to an increase in the prevalence rate.

This reasoning can be comprehended through the study by Berry et al.,^33^ who evaluated the reduction in mortality due to breast cancer by means of seven mathematical models. They concluded that both screening and treatment influenced the reduction in mortality, but they were unable to establish the significance of each of these actions separately, with regard to their impact on breast cancer mortality.^33^

The prevalence rate in Goiânia over the 15-year period was 127/100,000 women. This rate is similar to what has been found by other authors^3,11^ who used the conventional model to estimate prevalence, with values of 125.9/100,000 and 106.2/100,000 in North America and Europe, respectively.^3^ Diverging from previous data, other studies have shown higher prevalence rates in Europe, ranging from 300/100,000 to 1230/100,000.^2,5-8^ The difference between these numbers and the previous ones possibly results from the methodology adopted for calculating the prevalence, since these studies used mathematical models that differed from those used by Pisani et al.^3^ and Parkin et al.^11^ The prevalence rate for breast cancer in the city of Goiânia increased significantly over the course of the study period. However, comparison of this rate with rates in other locations must be done with caution, since the methodology for calculating the prevalence rate differs considerably among the various studies on this topic.

Another hypothesis that may explain the differences between the rates is the socioeconomic situations of different countries, since it has been demonstrated that in developed countries, the breast cancer prevalence rate is higher than it is in developing countries.^5^ Use of different periods and/or populations may have influenced the differences between prevalence rates among different authors.^2,4-8^

In the future, it is possible that there may be reductions in breast cancer prevalence in some parts of the world, due to greater primary prevention of breast cancer, including bilateral prophylactic mastectomy^34-36^ and chemoprevention through the use of tamoxifen or raloxifene.^37^ Another indicator of possible reductions can be seen in specific locations such as the United States, where there was a marked reduction in breast cancer incidence in the year 2003, possibly related to a reduction in hormone therapy prescription.^38,39^

## CONCLUSIONS

Thus, we conclude that the prevalence rate for breast cancer increased significantly in relation to the incidence rate, thereby demonstrating that opportunistic screening and improvements in diagnosis and treatment have been increasing life expectancy among women diagnosed with breast cancer in the city of Goiânia.
